# SARS-CoV-2 detection with CRISPR diagnostics

**DOI:** 10.1038/s41421-020-0174-y

**Published:** 2020-05-19

**Authors:** Lu Guo, Xuehan Sun, Xinge Wang, Chen Liang, Haiping Jiang, Qingqin Gao, Moyu Dai, Bin Qu, Sen Fang, Yihuan Mao, Yangcan Chen, Guihai Feng, Qi Gu, Ruiqi Rachel Wang, Qi Zhou, Wei Li

**Affiliations:** 1 State Key Laboratory of Stem Cell and Reproductive Biology, Institute of Zoology, Chinese Academy of Sciences, Beijing, 100101 China; 2 Institute for Stem Cell and Regeneration, Chinese Academy of Sciences, Beijing, 100101 China; 3 University of Chinese Academy of Sciences, Beijing, 100049 China

**Keywords:** Biological techniques, Molecular biology

Dear Editor,

The novel coronavirus (CoV) disease termed COVID-19 (coronavirus disease-19) caused by SARS-CoV-2 (severe acute respiratory syndrome coronavirus-2)^1^ is causing a massive pandemic worldwide, threatening public health systems across the globe. During this ongoing COVID-19 outbreak, nucleic-acid detection has played an important role in early diagnosis^2^. To date, four protocols based on CRISPR for detecting SARS-CoV-2 have been published^3–6^. Using lateral flow protocols, RNA samples harboring more than 1 × 10^4^–1 × 10^5^ copies/mL (SHERLOCK) or 1 × 10^4^ copies/mL (DETECTR) can be detected within 1 hour. In addition to these reported efforts, we have also established a SARS-CoV-2 detection protocol based on our previously reported platform—CDetection (Cas12b-mediated DNA detection)^7^. By combining sample treatment protocols and nucleic-acid amplification methods with CDetection, we have established an integrated viral nucleic-acid detection platform—CASdetec (CRISPR-assisted detection). The detection limit of CASdetec for SARS-CoV-2 pseudovirus is 1 × 10^4^ copies/mL, with no cross-reactivity observed. Here, we present our assay design and optimization process, which could provide guidance for future CRISPR-based nucleic-acid detection assay development and optimization.

To optimize the output of fluorescence signal, we designed and synthesized poly-T fluorescence-quenchers of varying nucleotide lengths, including 4 nt, 5 nt, 7 nt, 12 nt, 17 nt, 22 nt, and 27 nt. Of all the lengths tried, the 7 nt poly-T reporter provided the highest signals in the shortest time (Supplementary Fig. S1a, b). Based on this observation, we applied the 7 nt poly-T reporter in later experiments.

According to the published SARS-CoV-2 whole-genome sequence^8^, we designed seven sgRNAs around the RdRp locus, as recommended by World Health Organization (WHO)^2^ (Supplementary Fig. S2a and Table S1). Owing to its high similarity to SARS-CoV, we ran initial experiments on both SARS-CoV-2 and SARS-CoV plasmids. According to fluorescence kinetics studies, sgRNA-3 stood out in not only being able to distinguish between the two similar coronaviruses but also being able to produce the most distinct fluorescence signal (Fig. 1a and Supplementary Fig. S2b–g).

**Fig. 1:**
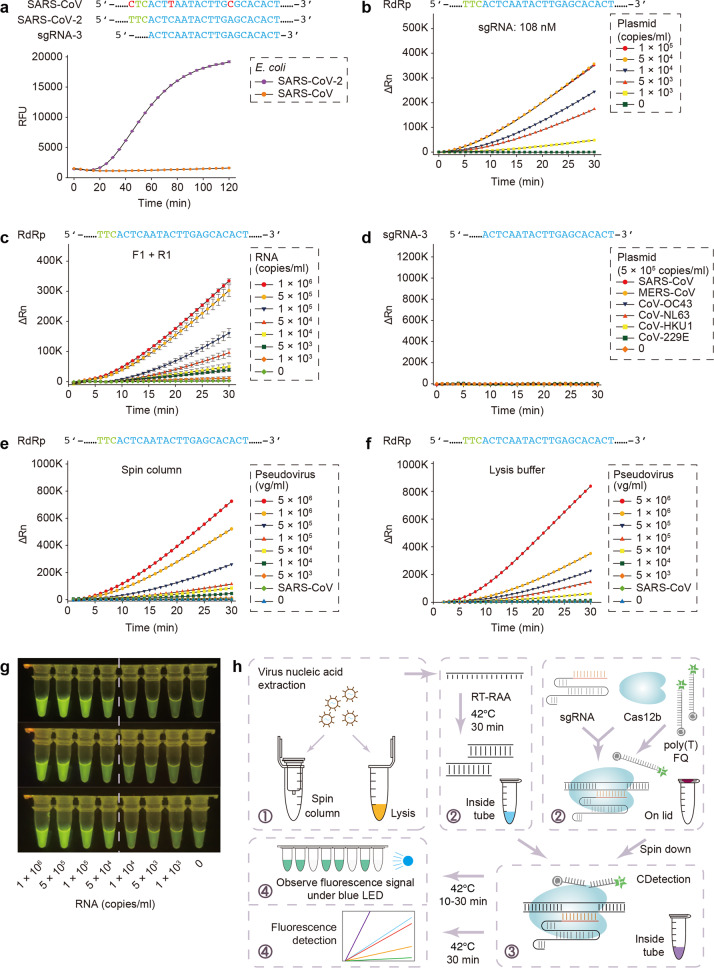
CASdetec used for SARS-CoV-2 detection. **a** Fluorescence kinetics of sgRNA-3 for RdRp detection. *E. coli* cells bearing Blunt-SARS-CoV-RdRp or Blunt-SARS-CoV-2-RdRp were pre-incubated at 95 °C for 10 min and used as templates for RAA and CDetection. PAM sequences are colored in green, protospacers are colored in blue, base pair mismatches are colored in red. Error bars indicate standard errors of the mean (s.e.m.), *n* = 3. RFU, relative fluorescence units. **b** Fluorescence kinetics of RdRp detection using 108 nM sgRNA-3. Plasmid bearing SARS-CoV-2-RdRp was serially diluted as shown in the legend. *n* = 2. ΔRn, ΔFluorescence, which refers to the Rn value of an experimental reaction minus the Rn value of the baseline signal generated by ABI 7500. **c** Fluorescence kinetics of F1- and R1-based RdRp detection. SARS-CoV-2-RdRp RNA was serially diluted as shown in the legend. Error bars indicate (s.e.m.), *n* = 3. **d** Evaluation of cross-reactivity. Plamids containing target RdRp region from six human epidemic coronaviruses were serially diluted as the shown in the legend. *n* = 2. **e** Detection of SARS-CoV-2 pseudovirus. Virus genome was extracted using the virus RNA extraction kit (spin column). SARS-CoV were diluted to 5 × 10^5^ copies/mL. *n* = 2. **f** Detection of SARS-CoV-2 pseudovirus. Virus was treated by direct lysis. SARS-CoV was diluted to 5 × 10^5^ copies/mL. *n* = 2. **g** CASdetec results could be directly observed under blue LED. 3 replicates of products from Fig. 1c were imaged upon blue LED illumination. **h** Schematics showing the workflow of CASdetec. Virus genome was extracted by kit or direct lysis. Target sequences were pre-amplified by isothermal amplification, followed was CDetection. Fluorescence signals were obtained either from fluorescence reader or direct observation under blue light.

As previously demonstrated, CRISPR is unable to detect any target DNA when there is <1–10 nM of amplification product within the reaction mix^7^. Hence, increasing the molecular collisions between CRISPR and target would be essential to improve sensitivity. We found out that increasing the sgRNA concentration by threefolds not only enhances the fluorescence signal and the signal-to-background ratio, but also increases the rate of reaction (Fig. 1b and Supplementary Fig. S3a, b).

Given that the average viral load in the plasma of SARS patients ranged from <1 to ~1000 copies per microliter^9^, or 1 × 10^3^–1 × 10^6^ copies/mL, nucleic-acid amplification techniques are needed to produce sufficient DNA for CRISPR-based DNA detection methods. Recombinase-aided amplification (RAA) can amplify substrates 10^10^ times at most (from 1 am to 10 nM) within 10–30 minutes at constant temperature between 37 °C and 42 °C, complementing the needs of CRISPR-based detection. Thus, we designed and screened RAA primers that matched our previously optimized sgRNA-3 (Supplementary Fig. S4a and Table S1). Based on our screenings, we found that by using the best primer pairs together with sgRNA-3, we can detect SARS-CoV-2 RNA samples containing 5 × 10^3^ copies/mL (Fig. 1c and Supplementary Fig. S4b, c).

In addition, we analyzed all SARS-CoV-2 sequences that have been uploaded to GISAID up till March 26th 2020. Out of 1792 sequences on GISAID, 1673 of them contained sequences matching our chosen primers and sgRNA. Only three of them have one mismatch to the forward primer and only two of them have one mismatch to the reverse primer (Supplementary Fig. S5), suggesting that our selected sgRNA and primers can be used for nearly all of the reported SARS-CoV-2 genomes. Meanwhile, we aligned the selected primers and sgRNAs to twelve typical human coronaviruses to evaluate their specificity, and found that none of the whole set of primers and sgRNA showed high similarity (Supplementary Fig. S6a–c).

The experiments above were conducted by executing RT-RAA nucleic-acid amplification and CDetection separately. However, it would be best to conduct both reactions within a single tube for convenience and, more importantly, to prevent aerosol contamination, which happens when the reaction mixture has to be exposed to the environment midway through the protocol. Hence, we tried to execute both the RT-RAA and CDetection concurrently within a single tube. However, the combination resulted in a drastic decrease in sensitivity (Fig. 1c and Supplementary Fig. S7). Therefore, in order to keep both the RT-RAA and CRISPR reactions within a single tube, we executed the RT-RAA reaction within the tube while keeping the CDetection reagents within the cap of the tube for 30 minutes, following which, the CDetection reagents were spun down into the tube for nucleic-acid detection, and the resultant reaction mixture was imaged for fluorescence.

To validate the specificity of our method for SARS-CoV-2 nucleic-acid detection, we tested our protocols against six coronaviruses known to cause respiratory diseases (SARS-CoV, MERS-CoV, CoV-HKU1, CoV-229E, CoV-OC43, and CoV-NL63). Consistent with alignment analysis (Supplementary Fig. S6a–c), no cross-reactivity with other endemic human coronavirus were detected (Fig. 1d). Our results suggested our set of sgRNA and primers showed high sensitivity and specificity.

However, viral genomes are packaged inside capsid protein and need to be released. Thus, to investigate the virus handling process, we produced pseudoviruses by packaging the target sequences of SARS-CoV-2, SARS-CoV, MERS-CoV into actual lentivirus particles (Supplementary Fig. S8). These pseudoviruses were diluted serially and treated with either virus genome extraction kits (spin column) or lysis buffer, respectively. Our results demonstrated that the spin column treatment gave a lower detection limit of 1 × 10^4^ copies/mL (Fig. 1e). On the other hand, the lysis buffer offered higher usability, but raised the detection limit to 5 × 10^4^ copies/mL (Fig. 1f). Owing to the difference in detection limit between spin column and lysis buffer, we suggest using the spin columns in hospitals, and using the lysis buffer for point-of-care testing (POCT).

To make CASdetec more amenable for POCT, we have also constructed a portable dark box containing a blue LED and demonstrated that the positive fluorescence signal generated from the protocol can be visualized upon illumination by a blue LED (Fig. 1g). The detection limit was deceased under blue LED compared with quantitative PCR instrument. Optimizing the extensity of excitation light and the sensitivity of optical detector may increase the detection limit of optical visualization. What’s more, a more impersonal readout method, such as taking photos followed by mobile software-mediated analyses, can be developed to analyze the optical results.

In conclusion, we have established a CASdetec platform, which consists of procedures including virus handling, nucleic-acid amplification, and CRISPR-based detection (Fig. 1h). CASdetec can detect pseudovirus samples with >1 × 10^4^ copies/mL, with no cross-reactivity to other endemic human coronaviruses. In addition, we optimized the workflow to run both reactions within one single tube without lid opening. This will thus prevent aerosol contamination and reduce the false positive rate. To guarantee the accuracy of CASdetec, efforts should be taken to avoid nucleic-acid aerosol contamination, including (1) conducting reagent preparation, sample treatment, amplification, and product analyzation in separate rooms and (2) being careful on handling samples and reagents to avoid contamination from touch.

## Supplementary information

Supplementary information

## Supplementary information

**Supplementary Information** accompanies the paper at (10.1038/s41421-020-0174-y).
