# Supplementary material for: SARS-CoV-2 detection with CRISPR diagnostics
Source: Cell Discov. 2020 May 19;6:34. doi: 10.1038/s41421-020-0174-y (PMC7235268; doi:10.1038/s41421-020-0174-y)
Supplement: Supplementary file 1 — Supplementary information [file 41421_2020_174_MOESM1_ESM.pdf]

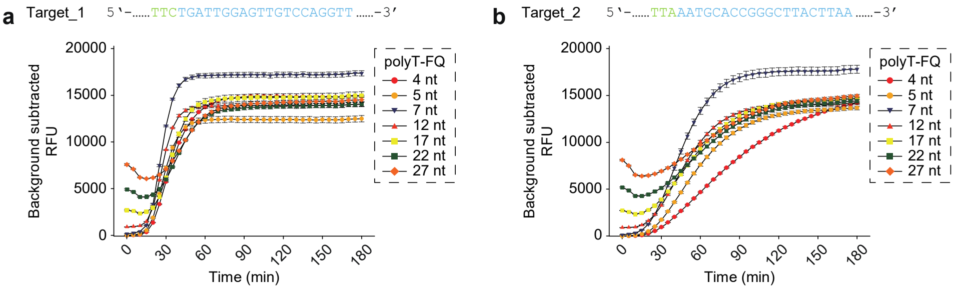

Supplementary Fig. S1 Optimization of reporter length. a-b Length preference of CDetection reporter. PAM sequences are colored in green, protospacers are colored in blue. Error bars indicate standard errors of the mean (s.e.m.)., n = 3. polyT-FQ, reporter made of T-homopolymer with FAM fluorophore and BHQ1 quencher.

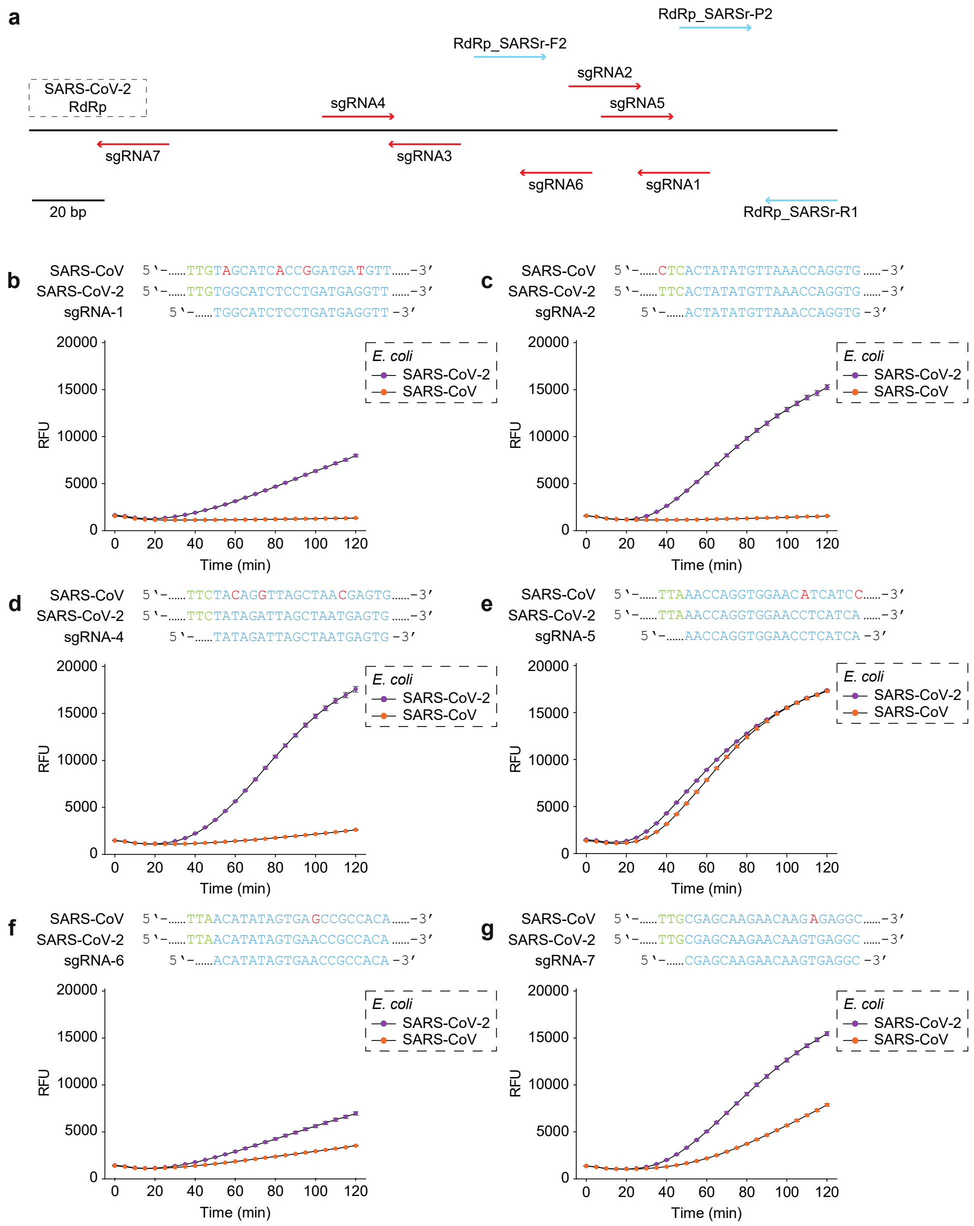

Supplementary Fig. S2 SgRNA selection for SARS-CoV-2 detection. a Schematics showing RdRp locus with sgRNAs, together with QPCR primers and probe disclosed by WHO. sgRNAs designed by ourselves are colored in red, QPCR primers and probe from WHO are colored in blue. bp, base pair. **b-g** Fluorescence kinetics of different sgRNAs for RdRp detection. *E. coli* cells bearing Blunt-SARS-CoV-RdRp or Blunt-SARS-CoV-2-RdRp were pre-incubated at 95°C for 10 min and used as templates for RAA and CDetection. PAM sequences are colored in green, protospacers are colored in blue, base pair mismatches are colored in red. Error bars indicate standard errors of the mean (s.e.m.)., n = 3. RFU, relative fluorescence units.

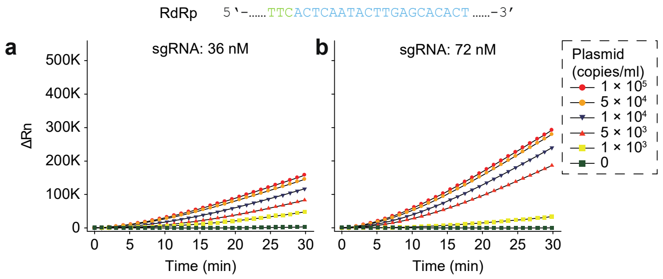

Supplementary Fig. S3 Optimization of sgRNA concentration. a, b Fluorescence kinetics of RdRp detection under 36 nM or 72 nM sgRNA-3. Plasmid bearing SARS-CoV-2-RdRp was serially diluted as shown in the legend. PAM sequences are colored in green, protospacers are colored in blue. $n = 2$. ΔRn, ΔFluorescence, which refers to the Rn value of an experimental reaction minus the Rn value of the baseline signal generated by ABI 7500.

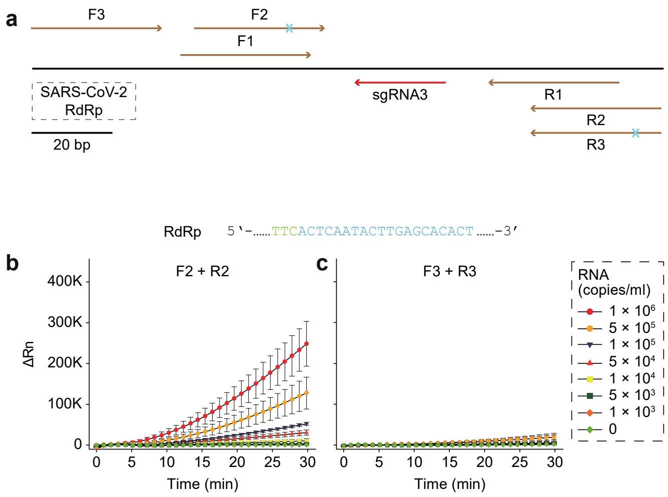

Supplementary Fig. S4 Primer selection for SARS-CoV-2 detection. a Schematics showing RdRp locus with RAA primers and sgRNA-3. The selected sgRNA-3 are colored in red, designed RAA primers are colored in brown. Blue cross indicated one base pair mismatch. bp, base pair. **b** Fluorescence kinetics of F2 and R2 based RdRp detection. SARS-CoV-2-RdRp RNA was serially diluted as shown in the legend. PAM sequences are colored in green, protospacers are colored in blue. Error bars indicate standard errors of the mean (s.e.m.), $n = 3$. ΔRn, ΔFluorescence, which refers to the Rn value of an experimental reaction minus the Rn value of the baseline signal generated by ABI 7500. **c** Fluorescence kinetics of F3 and R3 based RdRp detection. SARS-CoV-2-RdRp RNA was serially diluted as the legend show. Error bars indicate s.e.m., $n = 3$.

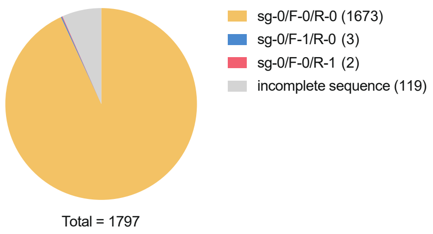

Supplementary Fig. S5 Conservation analysis of selected sgRNA and primers. Sequence alignment of 1797 reported SARS-CoV-2 sequences till March, 26th, 2020. Sg-0/F-0/R-0, genomes which have no mismatches to sgRNA-3 or F1 or R1. Sg-0/F-1/R-0, genomes which have no mismatches to sgRNA-3 or R1, but 1 mismatch to F1. Sg-0/F-0/R-1, genomes which have no mismatches to sgRNA-3 or F1, but 1 mismatch to R1. Incomplete sequence, incomplete sequencing results which was missed in the sgRNA-3, F1 and R1 region.

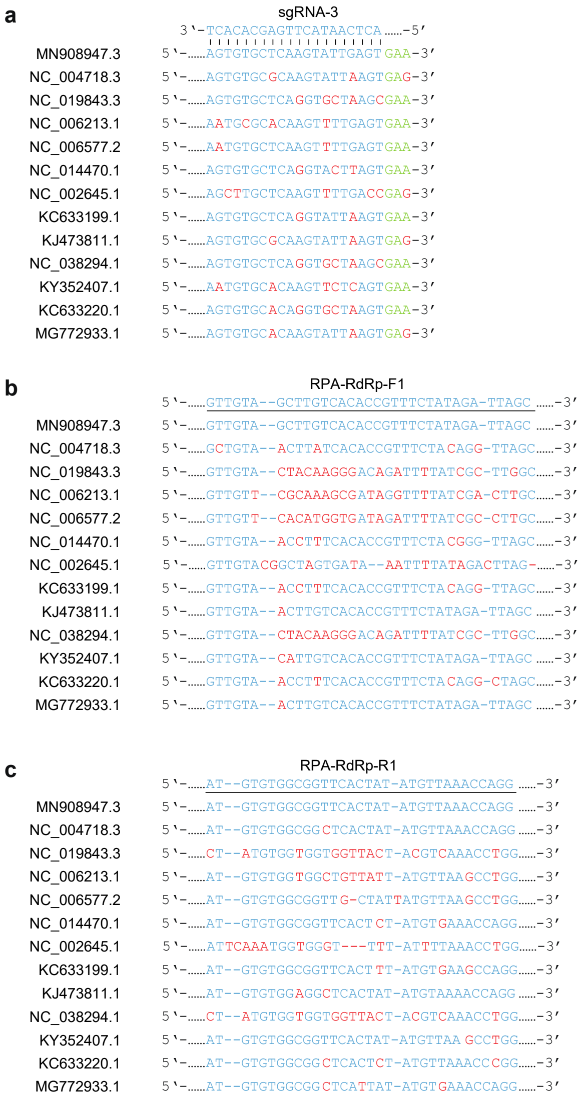

Supplementary Fig. S6 Specificity analysis of selected sgRNA and primers. a Sequence alignment of selected sgRNA among typical coronavirus sequences. PAM sequences are colored in green, protospacers are colored in blue, mismatched bases are colored in red. **b** Sequence alignment of selected forward primer among typical coronavirus sequences. **c** Sequence alignment of selected reverse primer among typical coronavirus sequences.

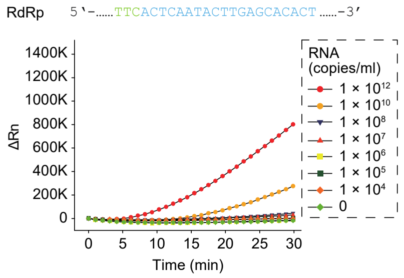

Supplementary Fig. S7 One-step detection of SARS-CoV-2 fragments. RAA and CDetection were combined into 1 step. SARS-CoV-2-RdRp RNA was serially diluted as the legend show. PAM sequences are colored in green, protospacers are colored in blue. $n = 2$. ΔRn, ΔFluorescence, which refers to the Rn value of an experimental reaction minus the Rn value of the baseline signal generated by ABI 7500.

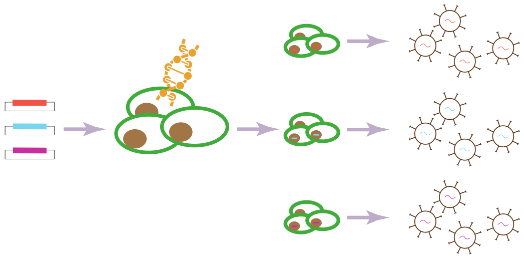

Supplementary Fig. S8 Construction of pseudoviruses. Schematics showing the procedure for pseudovirus packaging. Lentivirus plasmids bearing target RdRp region of SARS-CoV-2, SARS-CoV or MERS-CoV were co-transfected together with helper plasmids into HEK293T cells. Lentiviruses were harvested from the media.

Supplementary Materials and Methods

Protein purification

AaCas12b proteins were purified by GenScript (New Jersey, USA). Briefly, BPK2014-AaCas12b-His_{10} was cloned into *E. coli* strain BL21 (λDE3) and expression was induced with IPTG at 15°C for 16 h. Cell pellets were resuspended with lysis buffer followed by sonication. Target protein was obtained by two-step purification using Ni column and Superdex 200 column. Purified AaCas12b proteins were dialyzed, concentrated and quantified using BCA Protein Assay Kit (Thermo Fisher Scientific, Massachusetts, USA).

Nucleic acid preparation

DNA oligos were commercially purchased (GenScript). Double-stranded DNA activators were obtained by PCR reaction and purified using Oligo Clean & Concentrator Kit (ZYMO Research, California, USA). In order to avoid false positive results caused by target strand (TS) ssDNA, we used non-target strand (NTS) ssDNA as PCR template. PCR primers and ssDNA templates were listed in Supplementary Table S1.

Guide RNAs were transcribed *in vitro* using HiScribe™ T7 High Yield RNA Synthesis Kit (New England Biolabs, Massachusetts, USA) and purified using MicroElute RNA Clean Up Kit (Omega Bio-tek, Georgia, USA). AaCas12b sgRNA (AasgRNA) templates for in vitro RNA transcription were PCR amplified using primers bearing a T7 promoter (Supplementary Table S1).

Target sequences were assembled by high-fidelity PCR as previously reported[1]. Briefly, DNA oligos were commercially purchased (GenScript) (Supplementary Table S1). PCR

products were ligated to pEASY-Blunt (TransGen Biotech, Beijing, China) and sequenced. Plasmids were extracted from *E. coli* clones bearing the right sequences.

SARS-CoV-2-RdRp RNA was transcribed *in vitro* using HiScribe™ T7 High Yield RNA Synthesis Kit (New England Biolabs) and purified using MicroElute RNA Clean Up Kit (Omega Bio-tek). SARS-CoV-2-RdRp RNA templates for *in vitro* RNA transcription were PCR amplified using primers bearing a T7 promoter (Supplementary Table S1).

Reverse-transcription recombinase aided amplification (RT-RAA) assays

Reverse-transcription recombinase aided amplification (RT-RAA) kit were purchased from Hangzhou ZC Bio-Sci&Tech Co, Ltd (Hangzhou, China) and used according to the manufacturer's protocol with addition of 120 U Murine RNase Inhibitor (Vazyme, Nanjing, China). The 50 μL RT-RAA reaction system containing varying amounts of DNA input was incubated in 42°C for 30 minutes. All RAA products were directly used in the 55 μL detection assay as mentioned below.

CDetection assays

Reporter length optimization were performed with 30 nM AaCas12b, 36 nM sgRNA, 40 nM activator, 200 nM custom synthesized homopolymer ssDNA FQ reporter (Supplementary Table S1) and NEBuffer™ 2.1 in a 20 μL reaction in a Corning® 384-well Polystyrene NBS Microplate. Reactions were incubated at 42°C for indicated time course in a fluorescence plate reader (BioTek Synergy 4, Vermont, USA) with fluorescent kinetics measured every 5 min (λ_{ex}=485 nm; λ_{em}=528 nm, transmission gain=61). The fluorescence results were analyzed by

SigmaPlot software.

RdRp detection assays were performed as follow. 6 ul templates were RT-RAA amplified with CDetection system on lid. CDetection system consisted of 30 nM AaCas12b, 108 nM sgRNA (unless otherwise indicated), 200 nM custom synthesized homopolymer ssDNA FQ reporter (Supplementary Table S1), 40 U Murine RNase Inhibitor (Vazyme), 10 mM Tris-HCl (pH 7.5), 10 mM $MgCl_2$ and 1 mM DTT in a 5 μL reaction. After finishing RT-RAA assay at 42°C for 30 minutes, CDetection was spun down and incubated at 42°C for 30 minutes in Applied Biosystems 7500 real-time PCR system (Thermo Fisher Scientific) with fluorescence kinetics measured every minute. ΔRn value were exported and analyzed by SigmaPlot software.

Production of pseudovirus

Blunt-SARS-CoV-2-RdRp, Blunt-SARS-CoV-RdRp, Blunt-MERS-CoV-RdRp plasmids were digested by BamHI (New England Biolabs) and XbaI (New England Biolabs), and ligated to lenti-CRISPR-V2 (a gift from Feng Zhang, Addgene plasmid #52961), to produce lenti-SARS-CoV-2-RdRp, lenti-SARS-CoV-RdRp and lenti-MERS-CoV-RdRp, respectively. Together with psPAX2 (a gift from Didier Trono, Addgene plasmid #12260) and pMD2.G (a gift from Didier Trono, Addgene plasmid #12259), lenti plasmids were co-transfected by lipofectamine LTX with Plus Reagent (Theromo Fisher Scientific) into HEK293T cells. 48 and 72 hours after transfection, medium was harvested, filtered and concentrated by Amicon® Ultra-15 Centrifugal Filter (Merck Millipore, Darmstadt, Germany). Titration was calculated by QPCR.

Preparation of lentivirus

To mimic practical virus detection, throat swab was added to virus transport medium (Youkang, Beijing, China) and the resultant liquid was used to dilute lentivirus for our following experiments.

QIAamp Viral RNA Mini Kit (50) (QIAGEN, Dusseldorf, Germany) was used in accordance to the manufacturer's protocol. RNA extraction was done using 140 μL of sample and 25 μL nuclease-free water was used for elution.

Samples were treated with DTT/EDTA and heating prior to detection. 83 mM DTT and 0.83 mM EDTA were added to sample and followed by 2-step inactivation of 50°C for 5 minutes and 64°C for 5 minutes using a dry heat block. This product was used for subsequent CASdetec reaction.

Statistical analysis

Statistical analyses were performed using Prism Software (GraphPad). For statistical comparison, Student's t test was employed. A value of $p < 0.05$ was considered significant.

References:

1 Li, G., Dong, B., Liu, Y., Li, C. & Zhang, L. Gene synthesis method based on overlap extension PCR and DNAWorks program. *Methods Mol Biol* **1073**, 9-17, doi:10.1007/978-1-62703-625-2_2 (2013).

Supplementary Table S1 Nucleic acids used in this study. The T7 promoter sequences are colored in green, and plasmid inserted sequences are colored in orange, respectively. Spacer sequences are underlined.

**FQ ssDNA reporter**	
polyT-FQ-4nt	FAM-TTTT-BHQ1
polyT-FQ-5nt	FAM-TTTTT-BHQ1
polyT-FQ-7nt	FAM-TTTTTTT-BHQ1
polyT-FQ-12nt	FAM-TTTTTTTTTTTT-BHQ1
polyT-FQ-17nt	FAM-TTTTTTTTTTTTTTTTT-BHQ1
polyT-FQ-22nt	FAM-TTTTTTTTTTTTTTTTTTTTTT-BHQ1
polyT-FQ-27nt	FAM-TTTTTTTTTTTTTTTTTTTTTTTTTTT-BHQ1
**Oligo**	
SARS-CoV-2-RdRp-1	GTTTATAGTGATGTAGAAAACCCTCACCTTATGGGTTGGG
SARS-CoV-2-RdRp-2	CATGGCTCTATCACATTTAGGATAATCCCAACCCATAAGGTGAGGGT
SARS-CoV-2-RdRp-3	GGATTATCCTAAATGTGATAGAGCCATGCCTAACATGCTTAGAAT
SARS-CoV-2-RdRp-4	GCAAGAACAAGTGAGGCCATAATTCTAAGCATGTTAGGCATGGCTCT
SARS-CoV-2-RdRp-5	TTAGAATTATGGCCTCACTTGTTCTTGCTCGCAAACATACAACGT
SARS-CoV-2-RdRp-6	GGTGTGACAAGCTACAACACGTTGTATGTTTGCGAGCAAGAACA
SARS-CoV-2-RdRp-7	ACAACGTGTTGTAGCTTGTCACACCGTTTCTATAGATTAGCTAATGAG
SARS-CoV-2-RdRp-8	CTCAATACTTGAGCACACTCATTAGCTAATCTATAGAAACGGTGTG
SARS-CoV-2-RdRp-9	AGCTAATGAGTGTGCTCAAGTATTGAGTGAAATGGTCATGTGTGG
SARS-CoV-2-RdRp-10	GTTTAACATATAGTGAACCGCCACACATGACCATTTCACTCAATAC
SARS-CoV-2-RdRp-11	GTGTGGCGGTTCACTATATGTTAAACCAGGTGGAACCTCATCAGG
SARS-CoV-2-RdRp-12	CATAAGCAGTTGTGGCATCTCCTGATGAGGTTCCACCTGGTTTAA
SARS-CoV-2-RdRp-13	TCAGGAGATGCCACAACTGCTTATGCTAATAGTGTTTTTAACATTT
SARS-CoV-2-RdRp-14	AAATGTTAAAAACACTATTAGCATAA
SARS-CoV-RdRp-1	GTTTACAGTGATGTAGAAACTCCACACCTTATGGGTTGGGATTATCCAAAATGTGAC
SARS-CoV-RdRp-2	GCATGTTAGGCATGGCTCTGTCACATTTTGGATAATCCCAACCCATAAGG
SARS-CoV-RdRp-3	GTGACAGAGCCATGCCTAACATGCTTAGGATAATGGCCTCTCTTGTTCTTGCTC
SARS-CoV-RdRp-4	TGTGATAAGTTACAGCAAGTGTTATGTTTGCGAGCAAGAACAAGAGAGGCCATTATCCT
SARS-CoV-RdRp-5	CGCAAACATAACACTTGCTGTAACTTATCACACCGTTTCTACAGGTTAGCTAACG
SARS-CoV-RdRp-6	CCATCTCACTTAATACTTGCGCACACTCGTTAGCTAACCTGTAGAAACGGTGTGA
SARS-CoV-RdRp-7	GAGTGTGCGCAAGTATTAAGTGAGATGGTCATGTGTGGCGGCTCACTATATG
SARS-CoV-RdRp-8	CACCGGATGATGTTCCACCTGGTTTAACATATAGTGAGCCGCCACACATGAC
SARS-CoV-RdRp-9	AAACCAGGTGGAACATCATCCGGTGATGCTACAACTGCTTATGCTAATAGTGTCTTTAA
SARS-CoV-RdRp-10	AAATGTTAAAGACACTATTAGCATAAGCAGTTGTAGC
MERS-CoV-RdRp-1	TTGTACAAAGATGTTGATAATCCGCATCTTATGGGTTGGGATTACCCTAAGTGTG
MERS-CoV-RdRp-2	GATTCTACACATATTAGGCATAGCTCTATCACACTTAGGGTAATCCCAACCCATAAGA
MERS-CoV-RdRp-3	GTGATAGAGCTATGCCTAATATGTGTAGAATCTTCGCTTCACTCATATTAGCTCGTAA

MERS-CoV-RdRp-4	CCTTGTAGTACAACAAGTGCCATGTTTACGAGCTAATATGAGTGAAGCGAAGATTCT
MERS-CoV-RdRp-5	CGTAAACATGGCACTTGTTGTACTACAAGGGACAGATTTTATCGCTTGGCAAATGAG
MERS-CoV-RdRp-6	CGCTTAGCACCTGAGCACACTCATTTGCCAAGCGATAAAATCTGTCCCT
MERS-CoV-RdRp-7	GAGTGTGCTCAGGTGCTAAGCGAATATGTTCTATGTGGTGGTGGTTACTACGTC
MERS-CoV-RdRp-8	CCGCTACTGGTACCTCCAGGTTTGACGTAGTAACCACCACCACATAGAAC
MERS-CoV-RdRp-9	AAACCTGGAGGTACCAGTAGCGGAGATGCCACCACTGCATATGCCAATAGTG
MERS-CoV-RdRp-10	AAATGTTAAAGACACTATTGGCATATGCAGTGGTGGC
CoV-HKU1-1	CTTATAAAGGATGTTGACAACCCTGTTCTTATGGGTTGGGATTATCCTAAATGTG
CoV-HKU1-2	GCAAAATATTTGGCATAGCACGATCACATTTAGGATAATCCCAACCCATAAGA
CoV-HKU1-3	GTGATCGTGCTATGCCAAATATTTTGCGTATTGTTAGTAGTTTAGTTTTGGCCC
CoV-HKU1-4	CCATGTGAACAACAAAATTCATGTTTGCGGGCCAAAACTAAACTACTAACAATACG
CoV-HKU1-5	CGCAAACATGAATTTTGTTGTTCACATGGTGATAGATTTTATCGCCTTGCGA
CoV-HKU1-6	ATAACTATTTCACTCAAAACTTGAGCACATTCATTCGCAAGGCGATAAAATCTATCACC
CoV-HKU1-7	GAATGTGCTCAAGTTTTGAGTGAAATAGTTATGTGTGGCGGTTGCTATTATG
CoV-HKU1-8	CACTGCTAGTACCACCAGGCTTAACATAATAGCAACCGCCACACATAAC
CoV-HKU1-9	AAGCCTGGTGGTACTAGCAGTGGTGATGCAACTACTGCTTTTGCTAATTCTGTTTTTAA
CoV-HKU1-10	ATATATTAAAAACAGAATTAGCAAAAGCAGTAGTTGC
CoV-OC43-1	GCCTTATTAAAGATGTTGACAATCCTGTACTTATGGGTTGGGATTATCCTAAG
CoV-OC43-2	GGTTTGGCATAGCACGATCACACTTAGGATAATCCCAACCCATAAGTAC
CoV-OC43-3	GTGTGATCGTGCTATGCCAAACCTACTACGTATTGTTAGTAGTTTGGTATTAGCCC
CoV-OC43-4	CGAACAACATGTCTCATGTTTTCGGGCTAATACCAAACTACTAACAATACGTA
CoV-OC43-5	CCCGAAAACATGAGACATGTTGTTCGCAAAGCGATAGGTTTTATCGACTTGCG
CoV-OC43-6	CAATTTCACTCAAAACTTGTGCGCATTCATTCGCAAGTCGATAAAACCTATCGCTTT
CoV-OC43-7	ATGAATGCGCACAAGTTTTGAGTGAAATTGTTATGTGTGGTGGCTGTTATTATGTTAAG
CoV-OC43-8	CACTACTAGTGCCACCAGGCTTAACATAATAACAGCCACCACACATAACAA
CoV-OC43-9	TTAAGCCTGGTGGCACTAGTAGTGGTGATGCAACTACTGCTTTTGCTAATTCAGTC
CoV-OC43-10	ATGTTAAAGACTGAATTAGCAAAAGCAGTAGTTGCAT
CoV-NL63-1	ATGAAACTTTTCTTGATTTTGCTTATTTTGCCCCTGGTTTCTTGCTTTTCT
CoV-NL63-2	GTAACATAGAAATACTAGCATTACTGTTACATGTAGAAAAGCAAGAAACCAGGGGCAAA
CoV-NL63-3	CATGTAACAGTAATGCTAGTATTTCTATGTTACAATTAGGTGTTCCTGATAACTCTT
CoV-NL63-4	GCAACAAACCTGTGACAATAGTTGAAGAGTTATCAGGAACACCTAATTGTAACAT
CoV-NL63-5	TCTTCAACTATTGTCACAGGTTTGTTGCCAGTCCATTGGATTTGTGCTAATC
CoV-NL63-6	CATTGGCTGGGTAAGTAGATGTGCTCTGATTAGCACAAATCCAATGGACTGG
CoV-NL63-7	AGCACATCTACTTACCCAGCCAATGGCTTTTTCTATATTGATGTCGGTAAACACCGTA

CoV-NL63-8	CACTATGGAGTGCAAAAGCACTACGGTGTTTACCGACATCAATATAGAAAAA
CoV-NL63-9	CGTAGTGCTTTTGCACTCCATAGTGGTTATTATGATGCTAACCAGTATTATATTTATCT
CoV-NL63-10	TAGTGAGATAAATATAATACTGGTTAGCATCATAATA
CoV-229E-1	GGTTCTCAAACAGTTCTAAGATGCGGTGATTGTTTACGCAGACCGATGTTGT
CoV-229E-2	CATGATCATAGGCGCACTTAGTGCACAACATCGGTCTGCGTAAACAATCA
CoV-229E-3	GCACTAAGTGCGCCTATGATCATGTGTTTGGCACTGATCATAAGTTCATTTTAGCTATT
CoV-229E-4	GATGTGTTACACACATATGGTGTAATAGCTAAAATGAACTTATGATCAGTGCCAAA
CoV-229E-5	GCTATTACACCATATGTGTGTAACACATCTGGCTGCAATGTAAATGACGTTAC
CoV-229E-6	CAGTAATAATTCAAACCTCCAAGATACAGTTTTGTAACGTCATTTACATTGCAGCCAGA
CoV-229E-7	AAACTGTATCTTGGAGGTTTGAATTATTACTGTGTAGACCACAAACCACATCT
CoV-229E-8	CCAGCTGAACACAGTGGGAATGAAAGATGTGGTTTGTGGTCTACACAGTA
CoV-229E-9	TCATTCCCACTGTGTTCAGCTGGTAATGTCTTTGGTTTGTACAAAAGTTCTGCTTTG
CoV-229E-10	TGGAACCCAAAGCAGAACTTTTGTACAAACCAAAGAC
**DNA**	
Target_1_NTS-100	AAACACTTACAGAAAGTTGTATTACCAGGTGGAAGGTTCTGATTGGAGTTGTCCAGGTTTTTGGCACGTTGAACAAATAATTGAACATCATGCATGAACA
Target_2_NTS-100	CGCCAGGGTTTTCCCAGTCACGACAAAATCATAAAGTTAAATGCACCGGGCTTACTTAACAGCTTTTCGCTTTGAATCCTGTGTGAAATTGTTATCCGCT
ds_activator_RdRp_1 (277bp)	GTTTATAGTGATGTAGAAAACCCTCACCTTATGGGTTGGGATTATCCTAAATGTGATAGAGCCATGCCTAACATGCTTAGAATTATGGCCTCACTTGTTCTTGCTCGCAAACATACAACGTGTTGTAGCTTGTCACACCGTTTCTATAGATTAGCTAATGAGTGTGCTCAAGTATTGAGTGAAATGGTCATGTGTGGCGGTTCACTATATGTTAAACCAGGTGGAACCTCATCAGGAGATGCCACAACTGCTTATGCTAATAGTGTTTTTAACATTT
T7-sgRNA-Target_1	TAATACGACTCACTATAGGGTCTAAAGGACAGAATTTTTCAACGGGTGTGCCAATGGCCACTTTCCAGGTGGCAAAGCCCGTTGAACTTCAAGCGAAGTGGCAC<u>TGATTGGAGTTGTCCAGGTT</u>
T7-sgRNA-Target_2	TAATACGACTCACTATAGGGTCTAAAGGACAGAATTTTTCAACGGGTGTGCCAATGGCCACTTTCCAGGTGGCAAAGCCCGTTGAACTTCAAGCGAAGTGGCAC<u>AATGCACCGGGCTTACTTAA</u>
T7-AasgRNA-RdRp-1	TAATACGACTCACTATAGGGTCTAAAGGACAGAATTTTTCAACGGGTGTGCCAATGGCCACTTTCCAGGTGGCAAAGCCCGTTGAACTTCAAGCGAAGTGGCAC<u>TGGCATCTCCTGATGAGGTT</u>
T7-AasgRNA-RdRp-2	TAATACGACTCACTATAGGGTCTAAAGGACAGAATTTTTCAACGGGTGTGCCAATGGCCACTTTCCAGGTGGCAAAGCCCGTTGAACTTCAAGCGAAGTGGCACAC<u>TATATGTTAAACCAGGTG</u>
T7-AasgRNA-RdRp-3	TAATACGACTCACTATAGGGTCTAAAGGACAGAATTTTTCAACGGGTGTGCCAATGGCCACTTTCCAGGTGGCAAAGCCCGTTGAACTTCAAGCGAAGTGGCAC<u>ACTCAATACTTGAGCACACT</u>
T7-AasgRNA-RdRp-4	TAATACGACTCACTATAGGGTCTAAAGGACAGAATTTTTCAACGGGTGTGCCAATGGCCACTTTCCAGGTGGCAAAGCCCGTTGAACTTCAAGCGAAGTGGCAC<u>TATAGATTAGCTAATGAGTG</u>

T7-AasgRNA-RdRp-5	TAATACGACTCACTATAGGGTCTAAAGGACAGAATTTTTCAACGGGTGTGCCAATGGCCACTTTCCAGGTGGCAAAGCCCGTTGAACTTCAAGCGAAGTGGCACAACCAGGTGGAACCTCATCA
T7-AasgRNA-RdRp-6	TAATACGACTCACTATAGGGTCTAAAGGACAGAATTTTTCAACGGGTGTGCCAATGGCCACTTTCCAGGTGGCAAAGCCCGTTGAACTTCAAGCGAAGTGGCACACATATAGTGAACCGCCACA
T7-AasgRNA-RdRp-7	TAATACGACTCACTATAGGGTCTAAAGGACAGAATTTTTCAACGGGTGTGCCAATGGCCACTTTCCAGGTGGCAAAGCCCGTTGAACTTCAAGCGAAGTGGCACCGAGCAAGAACAAGTGAGGC
**Primers**	
Target_1-F	AAACACTTACAGAAAGTTGTATTACCAGGT
Target_1-R	TGTTCATGCATGATGTTCAATTATTTGTTC
Target_2-F	CGCCAGGGTTTTCCCAGTCACGAC
Target_2-R	AGCGGATAACAATTTCACACAGGA
RPA-RdRp-F0	GTTTATAGTGATGTAGAAAACCCTCACCTTAT (used in Fig. 1a, Fig. S2b-h)
RPA-RdRp-R0	AAATGTTAAAAACACTATTAGCATAAGCAGTT (used in Fig. 1a, Fig. S2b-h)
RPA-RdRp-F1	GTTGTAGCTTGTCACACCGTTTCTATAGATTAGC
RPA-RdRp-R1	CCTGGTTTAACATATAGTGAACCGCCACACAT
RPA-RdRp-F2	TTGTAGCTTGTCACACCGTTTATATAGATTAG
RPA-RdRp-R2	CACCTGGTTTAACATATAGTGAACCGCCACACA
RPA-RdRp-F3	TTAGAATTATGGCCTCACTTGTTCTTGCTC
RPA-RdRp-R3	CACCTGGATTAACATATAGTGAACCGCCACACA
T7-AasgRNA-F	TAATACGACTCACTATAGGGTCTAAAGGACAGAATTTTTCAACGGGTG
T7-sgRNA-Target_1-R	AACCTGGACAACTCCAATCAGTGCCACTTCGCTTGAAGTTCA
T7-sgRNA-Target_2-R	TTAAGTAAGCCCGGTGCATTGTGCCACTTCGCTTGAAGTTCA
T7-sgRNA-RdRp-1-R	AACCTCATCAGGAGATGCCAGTGCCACTTCGCTTGAAGTTCA
T7-sgRNA-RdRp-2-R	CACCTGGTTTAACATATAGTGTGCCACTTCGCTTGAAGTTCA
T7-sgRNA-RdRp-3-R	AGTGTGCTCAAGTATTGAGTGTGCCACTTCGCTTGAAGTTCA
T7-sgRNA-RdRp-4-R	CACTCATTAGCTAATCTATAGTGCCACTTCGCTTGAAGTTCA
T7-sgRNA-RdRp-5-R	TGATGAGGTTCCACCTGGTTGTGCCACTTCGCTTGAAGTTCA
T7-sgRNA-RdRp-6-R	TGTGGCGGTTCACTATATGTGTGCCACTTCGCTTGAAGTTCA
T7-sgRNA-RdRp-7-R	GCCTCACTTGTTCTTGCTCGGTGCCACTTCGCTTGAAGTTCA
QPCR-lenti-F	GGACGTCCTTCTGCTACGTC(used for determination of pseudovirus titration)
QPCR-lenti-R	GAGATCCGACTCGTCTGAGG(used for determination of pseudovirus titration)
**AasgRNA**	
AasgRNA-Target_1	GUCUAAAGGACAGAAUUUUUCAACGGGUGUGCCAAUGGCCACUUUCCAGGUGGCAAAGCCCGUUGAACUUCAAGCGAAGUGGCACUGAUUGGAGUUGUCCAGGUU
AasgRNA-Target_2	GUCUAAAGGACAGAAUUUUUCAACGGGUGUGCCAAUGGCCACUUUCCAGGUGGCAAAGCCCGUUGAACUUCAAGCGAAGUGGCACAAUGCACCGGGCUUACUUAA
AasgRNA-RdRp-1	GUCUAAAGGACAGAAUUUUUCAACGGGUGUGCCAAUGGCCACUUUCCAGGUGGCAAAGCCCGUUGAACUUCAAGCGAAGUGGCACUGGCAUCUCCUGAUGAGGUU
AasgRNA-RdRp-2	GUCUAAAGGACAGAAUUUUUCAACGGGUGUGCCAAUGGCCACUUUCCAGGUGGCAAAGCCCGUUGAACUUCAAGCGAAGUGGCACUAUAUGUUAAACCAGGUG
AasgRNA-RdRp-3	GUCUAAAGGACAGAAUUUUUCAACGGGUGUGCCAAUGGCCACUUUCCAGGUGGCAAAGCCCGUUGAACUUCAAGCGAAGUGGCACACUCAAUACUUGAGCACACU
AasgRNA-RdRp-4	GUCUAAAGGACAGAAUUUUUCAACGGGUGUGCCAAUGGCCACUUUCCAGGUGGCAAAGCCCGUUGAACUUCAAGCGAAGUGGCACUAUAGAUUAGCUAAUGAGUG

AasgRNA-RdRp-5	GUCUAAAGGACAGAAUUUUUCAACGGGUGUGCCAAUGGCCACUUUCCAGGUGGCA<br>AAGCCCGUUGAACUUCAAGCGAAGUGGCACAACCAGGUGGAACCUCAUCA
AasgRNA-RdRp-6	GUCUAAAGGACAGAAUUUUUCAACGGGUGUGCCAAUGGCCACUUUCCAGGUGGCA<br>AAGCCCGUUGAACUUCAAGCGAAGUGGCACACAUAUAGUGAACCGCCACA
AasgRNA-RdRp-7	GUCUAAAGGACAGAAUUUUUCAACGGGUGUGCCAAUGGCCACUUUCCAGGUGGCA<br>AAGCCCGUUGAACUUCAAGCGAAGUGGCACCGAGCAAGAACAAGUGAGGC
**pEASY-Blunt plasmid**	
pEASY-Blunt-SARS-CoV-2-RdRp	...ATATCTGCAGAATTGCCCTTGTTTATAGTGATGTAGAAAACCCTCACCTTAT<br>GGGTTGGGATTATCCTAAATGTGATAGAGCCATGCCTAACATGCTTAGAATTATG<br>GCCTCACTTGTTCTTGCTCGCAAACATACAACGTGTTGTAGCTTGTCACACCGTT<br>TCTATAGATTAGCTAATGAGTGTGCTCAAGTATTGAGTGAAATGGTCATGTGTGG<br>CGGTTCACTATATGTTAAACCAGGTGGAACCTCATCAGGAGATGCCACAACTGCT<br>TATGCTAATAGTGTTTTTAACATTTAAGGGCAATTCCAGCACACT
pEASY-Blunt-SARS-CoV-RdRp	...ATATCTGCAGAATTGCCCTTGTTTACAGTGATGTAGAAACTCCACACCTTAT<br>GGGTTGGGATTATCCAAAATGTGACAGAGCCATGCCTAACATGCTTAGGATAATG<br>GCCTCTCTTGTTCTTGCTCGCAAACATAACACTTGCTGTAACTTATCACACCGTT<br>TCTACAGGTTAGCTAACGAGTGTGCGCAAGTATTAAGTGAGATGGTCATGTGTGG<br>CGGCTCACTATATGTTAAACCAGGTGGAACATCATCCGGTGATGCTACAACTGCT<br>TATGCTAATAGTGTCTTTAACATTTAAGGGCAATTCCAGCACACT
pEASY-Blunt-MERS-CoV-RdRp	...ATATCTGCAGAATTGCCCTTAAATGTTAAAGACACTATTGGCATATGCAGTG<br>GTGGCATCTCCGCTACTGGTACCTCCAGGTTTGACGTAGTAACCACCACCACATA<br>GAACATATTCGCTTAGCACCTGAGCACACTCATTTGCCAAGCGATAAAATCTGTC<br>CCTTGTAGTACAACAAGTGCCATGTTTACGAGCTAATATGAGTGAAGCGAAGATT<br>CTACACATATTAGGCATAGCTCTATCACACTTAGGGTAATCCCAACCCATAAGAT<br>GCGGATTATCAACATCTTTGTACAAATTCCAGCACACTGGCGGCC...
pEASY-Blunt-CoV-HKU1-RdRp	...ATATCTGCAGAATTGCCCTTATATATTAAAAACAGAATTAGCAAAAGCAGTA<br>GTTGCATCACCACTGCTAGTACCACCAGGCTTAACATAATAGCAACCGCCACACA<br>TAACTATTTCACTCAAAACTTGAGCACATTCATTCGCAAGGCGATAAAATCTATC<br>ACCATGTGAACAACAAAATTCATGTTTGCGGGCCAAAACTAAACTACTAACAATA<br>CGCAAAATATTTGGCATAGCACGATCACATTTAGGATAATTCCAGCACACTGGCG<br>GCCGT...
pEASY-Blunt-CoV-OC43-RdRp	...ATATCTGCAGAATTGCCCTTATGTTAAAGACTGAATTAGCAAAAGCAGTAGT<br>TGCATCACCACTACTAGTGCCACCAGGCTTAACATAATAACAGCCACCACACATA<br>ACAATTTCACTCAAAACTTGTGCGCATTCATTCGCAAGTCGATAAAACCTATCGC<br>TTTGCGAACAACATGTCTCATGTTTTCGGGCTAATACCAAACTACTAACAATACG<br>TAGTAGGTTTGGCATAGCACGATCACACTTAGGATAATCCCAACCCATAAGTACA<br>GGATTGTCAACATCTTTAATAAGGCAAGGGCAATTCCAGCACACT...
pEASY-Blunt-CoV-NL63-RdRp	...ATATCTGCAGAATTGCCCTTTAGTGAGATAAATATAATACTGGTTAGCATCA<br>TAATAACCACTATGGAGTGCAAAAGCACTACGGTGTTTACCGACATCAATATAGA<br>AAAAGCCATTGGCTGGGTAAGTAGATGTGCTCTGATTAGCACAAATCCAATGGAC<br>TGGCAACAAACCTGTGACAATAGTTGAAGAGTTATCAGGAACACCTAATTGTAAC<br>ATAGAAATACTAGCATTACTGTTACATGTAGAAAAGCAAGAAACCAGGGGCAAAA<br>TAAGCAAAATCAAGAAAAGTTTCATAAGGGCAATTCCAGCACACT...
pEASY-Blunt-CoV-229E-RdRp	...ATATCTGCAGAATTGCCCTTTGGAACCCAAAGCAGAACTTTTGTACAAACCA<br>AAGACATTACCAGCTGAACACAGTGGGAATGAAAGATGTGGTTTGTGGTCTACAC<br>AGTAATAATTCAAACCTCCAAGATACAGTTTTGTAACGTCATTTACATTGCAGCC<br>AGATGTGTTACACACATATGGTGTAATAGCTAAAATGAACTTATGATCAGTGCCA

	AACACATGATCATAGGCGCACTTAGTGCACAACATCGGTCTGCGTAAACAATCAC<br>CGCATCTTAGAACTGTTTGAGAACCAAGGGCAATTCCAGCACACT...
**RNA**	
SARS-CoV-2-RdRp_RNA	GUUUAUAGUGAUGUAGAAAACCCUCACCUUAUGGGUUGGGAUUAUCCUAAAUGUG<br>AUAGAGCCAUGCCUAACAUGCUUAGAAUUAUGGCCUCACUUGUUCUUGCUCGCAA<br>ACAUACAACGUGUUGUAGCUUGUCACACCGUUUCUAUAGAUUAGCUAAUGAGUGU<br>GCUCAAGUAUUGAGUGAAAUGGUCAUGUGUGGCGGUUCACUAUAUGUUAAACCAG<br>GUGGAACCUCAUCAGGAGAUGCCACAACUGCUUAUGCUAAUAGUGUUUUUAACAU<br>UU
SARS-CoV-RdRp_RNA	GUUUACAGUGAUGUAGAAACUCCACACCUUAUGGGUUGGGAUUAUCCAAAAUGUG<br>ACAGAGCCAUGCCUAACAUGCUUAGGAUAAUGGCCUCUCUUGUUCUUGCUCGCAA<br>ACAUAACACUUGCUGUAACUUAUCACACCGUUUCUACAGGUUAGCUAACGAGUGU<br>GCGCAAGUAUUAAGUGAGAUGGUCAUGUGUGGCGGCUCACUAUAUGUUAAACCAG<br>GUGGAACAUCAUCCGGUGAUGCUACAACUGCUUAUGCUAAUAGUGUCUUUAACAU<br>UU
MERS-CoV-RdRp_RNA	AAAUGUUAAAGACACUAUUGGCAUAUGCAGUGGUGGCAUCUCCGCUACUGGUACC<br>UCCAGGUUUGACGUAGUAACCACCACCACAUAGAACAUAUUCGCUUAGCACCUGA<br>GCACACUCAUUUGCCAAGCGAUAAAAUCUGUCCCUUGUAGUACAACAAGUGCCAU<br>GUUUACGAGCUAAUAUGAGUGAAGCGAAGAUUCUACACAUAUUAGGCAUAGCUCU<br>AUCACACUUAGGGUAAUCCCAACCCAUAAGAUGCGGAUUAUCAACAUCUUUGUAC<br>AA
CoV-HKU1-RdRp_RNA	AUAUAUUAAAAACAGAAUUAGCAAAAGCAGUAGUUGCAUCACCACUGCUAGUACC<br>ACCAGGCUUAACAUAAUAGCAACCGCCACACAUAACUAUUUCACUCAAAACUUGA<br>GCACAUUCAUUCGCAAGGCGAUAAAAUCUAUCACCAUGUGAACAACAAAAUUCAU<br>GUUUGCGGGCCAAAACUAAACUACUAACAAUACGCAAAAUAUUUGGCAUAGCACG<br>AUCACAUUUAGGAUAAU
CoV-OC43-RdRp_RNA	AUGUUAAAGACUGAAUUAGCAAAAGCAGUAGUUGCAUCACCACUACUAGUGCCAC<br>CAGGCUUAACAUAAUAACAGCCACCACACAUAACAAUUUCACUCAAAACUUGUGC<br>GCAUUCAUUCGCAAGUCGAUAAAACCUAUCGCUUUGCGAACAACAUGUCUCAUGU<br>UUUCGGGCUAAUACCAAACUACUAACAAUACGUAGUAGGUUUGGCAUAGCACGAU<br>CACACUUAGGAUAAUCCCAACCCAUAAGUACAGGAUUGUCAACAUCUUUAAUAAG<br>GC
CoV-NL63-RdRp_RNA	UAGUGAGAUAAAUAUAAUACUGGUUAGCAUCAUAAUAACCACUAUGGAGUGCAAA<br>AGCACUACGGUGUUUACCGACAUCAAUAUAGAAAAAGCCAUUGGCUGGGUAAGUA<br>GAUGUGCUCUGAUUAGCACAAAUCCAAUGGACUGGCAACAAACCUGUGACAAUAG<br>UUGAAGAGUUAUCAGGAACACCUAAUUGUAACAUAGAAAUACUAGCAUUACUGUU<br>ACAUGUAGAAAAGCAAGAAACCAGGGGCAAAAUAAGCAAAAUCAAGAAAAGUUUC<br>AU
CoV-229E-RdRp_RNA	UGGAACCCAAAGCAGAACUUUUGUACAAACCAAAGACAUUACCAGCUGAACACAG<br>UGGGAAUGAAAGAUGUGGUUUGUGGUCUACACAGUAAUAAUUCAAACCUCCAAGA<br>UACAGUUUUGUAACGUCAUUUACAUUGCAGCCAGAUGUGUUACACACAUAUGGUG<br>UAAUAGCUAAAAUGAACUUAUGAUCAGUGCCAAACACAUGAUCAUAGGCGCACUU<br>AGUGCACAACAUCGGUCUGCGUAAACAAUCACCGCAUCUUAGAACUGUUUGAGAA<br>CC
**Lentivirus plasmid**	
Lenti-SARS-CoV-2-RdRp	...AGTGTGCTGGAATTGCCCTTGTTTATAGTGATGTAGAAAACCCTCACCTTAT<br>GGGTTGGGATTATCCTAAATGTGATAGAGCCATGCCTAACATGCTTAGAATTATG<br>GCCTCACTTGTTCTTGCTCGCAAACATACAACGTGTTGTAGCTTGTCACACCGTT

	TCTATAGATTAGCTAATGAGTGTGCTCAAGTATTGAGTGAAATGGTCATGTGTGG<br>CGGTTCACTATATGTTAAACCAGGTGGAACCTCATCAGGAGATGCCACAACTGCT<br>TATGCTAATAGTGTTTTTAACATTTAAGGGCAATTCTGCAGATAT...
Lenti-SARS-CoV-RdRp	...ATATCTGCAGAATTGCCCTTGtttacagtgatgtagaaactccacaccttat<br>gggttgggattatccaaaatgtgacagagccatgcctaacatgcttaggataatg<br>gcctctcttgttcttgctcgcaaacataacacttgctgtaacttatcacaccgtt<br>tctacaggttagctaacgagtgtgcgcaagtattaagtgagatggtcatgtgtgg<br>cggctcactatatgttaaaccaggtggaacatcatccggtgatgctacaactgct<br>tatgctaatagtgtctttaacatttAAGGGCAATTCCAGCACACT...
Lenti-MERS-CoV-RdRp	...AACACAGGACCGGTTCTAGAttgtacaaagatgttgataatccgcatcttat<br>gggttgggattaccctaagtgtgatagagctatgcctaatatgtgtagaatcttc<br>gcttcactcatattagctcgtaaacatggcacttgttgtactacaagggacagat<br>tttatcgcttggcaaatgagtgtgctcaggtgctaagcgaatatgttctatgtgg<br>tggtggttactacgtcaaacctggaggtaccagtagcggagatgccaccactgca<br>tatgccaatagtgtctttaacatttAAGGGCAATTCTGCAGATAT...
